# Using community conversations to integrate violence screening and referrals into HIV care for young women living with HIV in Lusaka, Zambia

**DOI:** 10.1002/jia2.26181

**Published:** 2023-10-11

**Authors:** Christina A. Laurenzi, Chipo Mutambo, Chanda Mwamba, Eugene Mupakile, Chuma Busakhwe, Agnes Ronan, Elona Toska

**Affiliations:** ^1^ Institute for Life Course Health Research, Department of Global Health, Stellenbosch University Tygerberg South Africa; ^2^ Paediatric‐Adolescent Treatment Africa Cape Town South Africa; ^3^ Centre for Social Science Research University of Cape Town Cape Town South Africa; ^4^ Kabangwe Creative Initiative Association (KCIA) Lusaka Zambia; ^5^ Department of Social Policy and Intervention University of Oxford Oxford UK

**Keywords:** adolescent girls and young women, community‐clinic partnerships, gender‐based violence, HIV service integration, participatory research, referrals

## 1 INTRODUCTION

Young women living with HIV (YWHIV) commonly experience overlapping vulnerabilities, especially in low‐resource, HIV‐endemic settings. They are likelier to experience violence, and have poorer antiretroviral adherence, HIV care disengagement and low viral suppression [1]. Despite strong prevention programming, HIV incidence among 15‐ to 24‐year‐olds in Zambia remains high, potentially exacerbated by the COVID‐19 pandemic [2]. Additionally, nearly two‐thirds of YWHIV in Zambia have experienced sexual violence from intimate partners [3]. HIV and violence commonly co‐occur in the context of harmful gender norms, early marriage, and power‐inequitable relationships, hindering access to healthcare services [4].

Violence screening at the health system interface can identify women at risk and link them to necessary services. While the World Health Organization recommends against universal screening for violence in healthcare settings, it provides guidance and training for integrating routine enquiry about violence into antenatal care [5]. However, more tailored screening approaches would help identify women requiring violence‐responsive services in settings with overburdened health systems and high rates of HIV and gender‐based violence (GBV) [5]. One clear evidence gap is designing and testing these integrated approaches.

Clinic‐community collaborations may be able to address this gap. Social stigma and norms surrounding HIV and GBV shape how they are experienced, reported and addressed in healthcare settings. For younger women, peer‐led approaches can facilitate less judgemental, more relatable interactions ‐ opening a critical entry point for screening and referrals, and improving post‐GBV service access. Yet, there is limited evidence about how to effectively upskill peer workers to respond to GBV [6], including for YWHIV. We aimed to explore how violence screening could be integrated into peer‐enhanced HIV care, using a clinic‐community collaborative model, for YWHIV.

In one low‐income community outside Lusaka, Zambia, we leveraged an existing partnership to co‐develop an integrated package, Screen & Support. This package included WHO's minimum requirements for violence screening (standard operating procedures, training, privacy, confidentiality and referral systems). It aimed to guide facility‐embedded youth peer supporters and their clinic‐based mentors to identify YWHIV—to whom they were already providing support—experiencing, or at risk of, violence, and link them to appropriate additional services.

To co‐design Screen & Support, we engaged diverse stakeholders in a series of community conversations (CCs) in October 2022 [7]. Applying qualitative, participatory methods within CCs, we discussed how to define GBV and contexts where GBV occurs; gauged knowledge and perceptions of GBV resources; and mapped referral pathways. Discussions were conducted primarily in Nyanja and Bemba, with some English, facilitated by a multi‐national research team.

We purposively invited 30 distinct individuals, including young women ages 15–24 and community members representing one or more stakeholder groups over the first 4 days of engagements (Table 1). All individuals provided informed consent, and adolescent girls under age 18 provided assent along with parent/guardian consent; consent processes included information about referral options given sensitive session content. YWHIV (*n* = 4) and those with unknown or negative HIV status (*n* = 8) were invited to participate. Serostatus‐neutral engagement allowed us to gather a broader perspective of how violence happens, how young women in particular experience it and how living with HIV may make experiences more complex; it also allowed us to mitigate potential stigma attached to this early‐stage work. Participants were recruited through our community‐based partner, KCIA. Participants attended separate sessions, based on their shared profile. A subset of participants (*n* = 10) was asked to return on a final day to collectively synthesize findings and map the next steps. The format encouraged participation and knowledge‐sharing: open‐ended questions were used to initiate discussions, alongside participatory exercises. Throughout these activities and sessions, stakeholders offered feedback on specific components of Screen & Support. The resulting package was designed to be integrated into HIV care, as YWHIV are a sub‐group at high risk of violence.

**Table 1:** Overview of community conversation participants and activities by session, October 2022

Day/Session	Participant profile	Number of participants	Participatory activities^a^
Day 1, Session 1	Young survivors of gender‐based violence (ages 15–24)	4	Journey mapping: Mapping out feelings, actions, responses and pain points, and opportunities for growth around GBV experiences (in pairs), followed by guided sharing with the group
Day 1, Session 2	Adolescent girls and young women living with HIV (ages 17–23)	4	Persona journeys: Creating visual personas of young women navigating a GBV incident (in pairs), followed by sharing with the group
Day 2, Session 3	Young married women (ages 21–24)	4	
Day 3, Session 4	Healthcare providers, including nurses, peer supporters and social welfare officers	10	Free‐listing barriers and facilitators to GBV reporting and care
Day 4, Session 5	Community and social leadership	8	
Day 5, Session 6	Multi‐stakeholder session (all participants were invited from previous days)	10	Review and consensus‐building on prior findings including collectively reviewing an anonymized‐by‐group list of key findings and themes; “How might we?” approach to solution‐finding linked to overall project aims, generating recommendations

^a^ Community conversation activities and data collection were granted ethical approval by the Centre for Social Science, University of Cape Town (CSSR 2022/03), ERES Converge Ethics Board, Lusaka, Zambia (2022‐Aug‐019), and the National Health Research Authority, Zambia (NHRA‐313/05/10/2022).

The emerging insights from these CCs, while context‐specific, are likely transferable to similar settings when considering how to integrate violence screening into HIV care for YWHIV.

## 2 EMERGING FINDINGS AND PROGRAMMING INSIGHTS

Our findings are organized into three sections following the post‐violence care cascade: screening, disclosure and reporting, and promising responses.

### 2.1 Screening

In considering ideal screening approaches, participants described the need for streamlined care, not adding to the duration of health facility visits. This was particularly relevant for young women who had small children and those with controlling spouses/partners. Participants suggested starting screening with rapport‐building, and asking general questions about young women's lives, rather than immediately beginning with structured questions. Young women specifically voiced preferences for less direct screening questions regarding specific types of violence experienced; they noted that violence screening during routine HIV service provision would need to be accompanied by a clear explanation and rationale to avoid patient discomfort. Additionally, limited space for confidential engagements was linked to concerns about their disclosing violence at community‐based health facilities being overheard by people who know them.

Participants indicated that screening should be conducted by a welcoming, non‐judgemental and knowledgeable individual. Peer supporters were noted by young women, community members and healthcare workers as a promising entry point—proximal enough to YWHIV, but also trusted for their foundational training and psychosocial support skills. Structuring expectations around peers’ time commitments, scope of work/roles, and supervision and support system emerged as key.

### 2.2 Disclosure and reporting

While conversations about ideal screening approaches signified that participants prioritized safe ways to disclose violence, processes for violence disclosure (confidential sharing) and reporting (requiring action) were often conflated in these sessions. Consequently, participants across groups described unwillingness to disclose violence as a major barrier, driven by social stigma and potential social and economic repercussions. Economic dependency on partners was a significant barrier to disclosing GBV, and to young women's agency; participants broadly described GBV as normalized within marriages. Importantly, HIV amplified these negative repercussions.

Formally reporting GBV was characterized as socially dangerous—for young married women, this could mean immediate income loss (if her husband was jailed), or divorce, which could lead to social isolation, ostracization and long‐term economic precarity. Cost was also a barrier for GBV survivors seeking support. Survivors faced a confusing system for reporting GBV, shunted between police, health facilities and other services. Participants described limited knowledge about free services. Additional costs for health services (e.g. pregnancy tests, or tests for HIV and sexually transmitted infections), transportation and required documentation were often borne by survivors or their families, deterring reporting. In cases where young survivors were assaulted by strangers or non‐intimate partners, participants described the informal process of settlements between families as a default, before police were involved.

### 2.3 Promising responses

Responding to these barriers and concerns, the final session's “how might we?” approach facilitated discussions about how to support broader integration, including: addressing harmful norms that drive GBV; identifying community structures for communication, advocacy and service coordination; and engaging young women directly in developing solutions. Participants advocated for stronger service networks and systems responsiveness among peer counsellors, GBV focal‐point staff at health facilities and community‐based stakeholders working in mental health, social care and justice.

## 3 RECOMMENDATIONS

Our findings elicit key considerations for others engaging in similar work. While many emerging themes were not HIV‐specific, proposed solutions showed where service integration could influence multiple improved outcomes. Layering violence screening into peer‐supported HIV care that encompasses clinic and community structures is likely to support more responsive, tailored care. Young women seeking HIV prevention services may also benefit from such violence screening services. Clear standard operating procedures for referral and support are critical, and can help connect YWHIV to additional services if no violence‐specific support is required. Peer‐delivered psychosocial support, such as the Ask‐Boost‐Connect‐Discuss programme [8], may provide adequate post‐GBV care in some cases. Existing tools, including the WHO LIVES training [9], could also be adapted and extended to enhance peer supporters’ skillsets. Finally, communication among key actors from multiple service sectors could enable frontline care providers to better access available services in their immediate and broader catchment areas. CCs and other approaches in the Clinic‐Community Collaboration adolescent toolkit can help initiate this engagement process [10], which can be invaluable for research and co‐designing multi‐sectoral partnerships.

## COMPETING INTERESTS

The authors declare no competing interests.

## AUTHORS’ CONTRIBUTIONS

CL and ET conceptualized the research project, with instrumental support from AR, C Mutambo and EM. Data collection (community conversation workshops) was facilitated by C Mwamba and supported closely by EM, C Mutambo and CL. Analysis was jointly conducted by C Mwamba, CL, C Mutambo, CB, EM, AR and ET. CL led the drafting of the manuscript, with contributions from C Mwamba and ET, and all authors approved the final version.

## FUNDING

This research was supported by Grand Challenges Canada Stars in Global Health (R‐ST‐POC‐2205‐52351).

## ETHICAL APPROVAL STATEMENT

Ethical approval for this project was granted by the Centre for Social Science, University of Cape Town (CSSR 2022/03), ERES Converge Ethics Board, Lusaka, Zambia (2022‐Aug‐019), and the National Health Research Authority, Zambia (NHRA‐313/05/10/2022).

## Data Availability

The data that support the findings of this study are available from the corresponding author upon reasonable request.
